# Supplementary material for: Fluorescence-Based Analysis of Noncanonical Functions of Aminoacyl-tRNA Synthetase-Interacting Multifunctional Proteins (AIMPs) in Peripheral Nerves
Source: Materials (Basel). 2019 Apr 1;12(7):1064. doi: 10.3390/ma12071064 (PMC6480683; doi:10.3390/ma12071064)
Supplement: Supplementary file 1 [file materials-12-01064-s001.pdf]

materials

Supplementary Materials: Fluorescence-Based Analysis of Noncanonical Functions of Aminoacyl-tRNA Synthetase-Interacting Multifunctional Proteins (AIMPs) in Peripheral Nerves

Muwoong Kim [1], Hyosun Kim [1,2], Dokyoung Kim [1], Chan Park [1], Youngbuhm Huh [1], Junyang Jung [1,*], Hyung-Joo Chung [3,*] and Na Young Jeong [4,*]

1. Department of Anatomy and Neurobiology, College of Medicine, Kyung Hee University, 26, Kyungheedae-ro, Dongdaemun-gu, Seoul 02447, Korea; qltnsdl9@khu.ac.kr (M.K.); hoysun0407@khu.ac.kr (H.K.); dkim@khu.ac.kr (D.K.); psychan@khu.ac.kr (C.P.); ybhuh@khu.ac.kr (Y.H.)
2. Department of Biomedical Science, Medical Research Center for Bioreaction to Reactive Oxygen Species and Biomedical Science Institute, Graduation School, Kyung Hee University, 26, Kyungheedae-ro, Dongdaemun-gu, Seoul 02447, Korea
3. Department of Anesthesiology and Pain Medicine, College of Medicine, Kosin University, 262, Gamcheon-ro, Seo-gu, Busan 49267, Korea
4. Department of Anatomy and Cell Biology, College of Medicine, Dong-A University, 32, Daesingongwon-ro, Seo-gu, Busan 49201, Korea

* Correspondence: jjung@khu.ac.kr (J.J.); dmadjejs@naver.com (H.-J.C.); jnyjjy@dau.ac.kr (N.Y.J.)

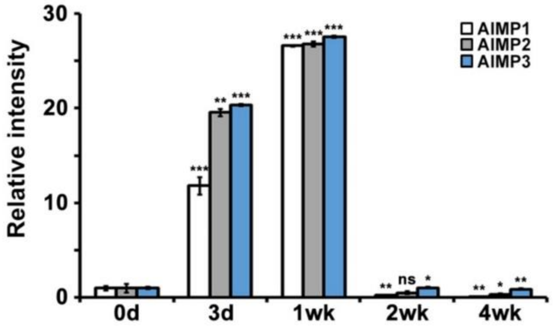

Figure S1. Relative intensities of mRNA expressions of aminoacyl-tRNA synthetase-interacting multifunctional proteins (AIMPs) using semi-PCR analysis were calculated by fold change. Fold changes of postinjury samples (3 days, 1 week, 2 weeks, 4 weeks) were derived by mRNA expression level of each sample divided by that of control (non-injured nerves) ($n = 3$, * $p < 0.05$, ** $p < 0.01$, *** $p < 0.001$, ns; $p > 0.05$). Glyceraldehyde-3-phosphate dehydrogenase (GAPDH) was used as loading control.

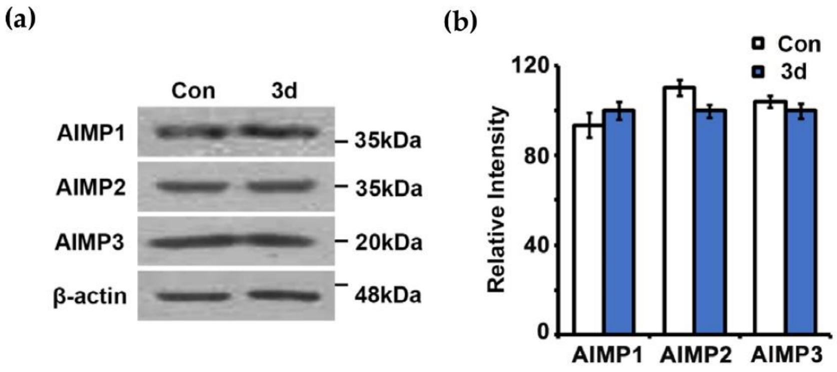

Figure S2. After peripheral nerve injury, there was no significant change of AIMPs expression in ventral horn of spinal cord. (**a**) 3 days after sciatic nerve injury, protein extracts (10 μg) from the ventral horn of spinal cord were evaluated by western blot. (**b**) Quantification of relative intensity of

AIMPs protein levels. Protein expression was relatively measured in the ventral horn compared with samples at 3 days after nerve injury.
